# Supplementary material for: Visible-to-mid-IR tunable frequency comb in nanophotonics
Source: Nat Commun. 2023 Oct 17;14:6549. doi: 10.1038/s41467-023-42289-0 (PMC10582254; doi:10.1038/s41467-023-42289-0)
Supplement: Supplementary file 1 — Supplementary Information [file 41467_2023_42289_MOESM1_ESM.pdf]

Supplemental Information: Visible-to-mid-IR tunable frequency comb in nanophotonics

Arkadev Roy[1*], Luis Ledezma[1,2*], Luis Costa[1], Robert Gray[1], Ryoto Sekine[1], Qiushi Guo[1], Mingchen Liu[1], Ryan M. Briggs[2], and Alireza Marandi[1†]

[1]Department of Electrical Engineering, California Institute of Technology, Pasadena, California 91125, USA

[2] Jet Propulsion Laboratory, California Institute of Technology, Pasadena, California 91109, USA

[*] These authors contributed equally to this work

[†]marandi@caltech.edu

Contents

1 System Parameters and dual-envelope simulation: 3

2 Single-envelope simulation and visible frequency comb: 5

3 Signal/Idler conversion efficiency: 10

4 Pump preparation/ Electro-optic frequency comb generation: 13

5 Quasi-sync pumping: 14

6 Estimating the cavity free spectral range: 16

7 Passive stability and frequency comb locking: 18

8 Mid-infrared absorption and it's near-infrared counterpart: 20

9 Spectral broadening/ Pulse compression in the degenerate OPO: 21

10 Carrier-Envelope offset frequency of the OPO frequency comb in the quasi-mode of operation: 23

11 Coherence verification using field cross-correlation technique: 24

12 Full system integration and a universal frequency comb source 25

13 Temperature tuning of the phase-matching curves 26

14 Pump wavelength tuning in doubly-resonant OPO 28

15 Future improvements and scope 31

1 System Parameters and dual-envelope simulation:

Here, we tabulate the system parameters that is used in the simulation.

Supplementary Table S1: Parameters for the LNOI OPO and pump pulse.

Symbol	Meaning	Value	Unit
$\lambda^{(b)}$	Pump Wavelength	1060	nm
L	PPLN Length	5	mm
u	Walk off/ GVM	0.174	ps/mm
T_p	Pump Pulse Width	1	ps
$\beta_2^{(a)}$	Signal GVD	4.21 x 10^{-5}	ps^2/mm
$\beta_3^{(a)}$	Signal TOD	-1.42 x 10^{-7}	ps^3/mm
$\beta_2^{(b)}$	Pump GVD	1.31 x 10^{-4}	ps^2/mm
$\beta_3^{(b)}$	Pump TOD	-4.93 x 10^{-6}	ps^3/mm
$\kappa^2 = \eta_0$	Normalized SHG efficiency	900%	$W^{-1}cm^{-2}$

The nonlinear interaction inside the PPLN waveguide (illustrated in Fig. S1) is governed by:

$$\frac{\partial a}{\partial z} = \left[-\frac{\alpha^{(a)}}{2} - i\frac{\beta_2^{(a)}}{2!}\frac{\partial^2}{\partial t^2} + \dots\right] a + \kappa a^* b \tag{S.1a}$$

$$\frac{\partial b}{\partial z} = \left[-\frac{\alpha^{(b)}}{2} - u\frac{\partial}{\partial t} - i\frac{\beta_2^{(b)}}{2!}\frac{\partial^2}{\partial t^2} + \dots\right] b - \kappa a^2 \tag{S.1b}$$

The evolution of the signal(a) and the pump(b) envelopes in the slowly varying envelope approximation are dictated by Eq. S.1(a) and Eq. S.1(b) respectively [1]. The effects of higher-order group velocity dispersions (GVD) β_2, β_3, group velocity mismatch (GVM) (u), and the back-conversion from the signal to the pump are included. The round-trip feedback is given by:

$$a^{(n+1)}(0,t) = \mathcal{F}^{-1}\left\{T(\omega)e^{i\bar{\phi}}\mathcal{F}\left\{a^{(n)}(L_{\text{cav}}, t)\right\}\right\} \tag{S.2a}$$

$$\bar{\phi} = \Delta\phi + \frac{l\lambda^{(a)}}{2c}(\omega - \omega_0) + \frac{\phi_2}{2!}(\omega - \omega_0)^2 + \dots \tag{S.2b}$$

Eq(S.2) takes into consideration the round-trip loss which is lumped into an aggregated out-coupling loss factor $1 - T(\omega)$ (considering the presence of two coupler regions in the OPO), the GVD ($\phi_2 = \beta_2^{(a)} L_{\text{fb}}$) of the feedback path and the detuning ($\Delta\phi$) ($\Delta\phi = \pi l + \phi_0$, l is the cavity length detuning in units of signal half-wavelengths in vacuum) of the circulating signal from the exact synchrony with respect to the pump. The effective second-order nonlinearity co-efficient (κ) is related to the SHG efficiency [1]. The round-trip number is denoted by n and the total cavity length by L_{cav}, with $L_{\text{fb}} = L_{\text{cav}} - L$. The equations are numerically solved by adopting the split-step Fourier algorithm.

Simulation of the non-degenerate OPO involves three coupled equations, one each for the signal, the idler, and the pump waves.

The difficulty of characterizing the Q-factor of our resonators (owing to the transmission nature of the adiabatic couplers and the unavailability of a tunable Cw source around 2 μm) prevented us from estimating the on-chip threshold for our OPOs. So, we assume certain parameters to estimate the

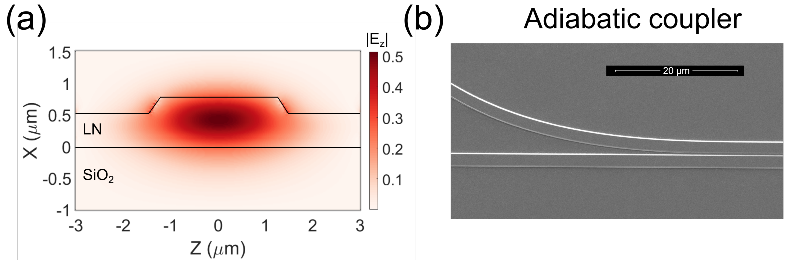

Supplementary Figure S1: Periodically poled lithium-niobate waveguide. a) The waveguides (dimensions: width of 2.5 μm, etch depth of 250 nm) support guided-modes in the mid-infrared corresponding to the idler wave, the electric field distribution (fundamental TE mode) of which is shown. b) The SEM image of the fabricated device showing the coupler region.

threshold power for the near-degenerate OPOs in the parameter space of second-harmonic generation efficiency and waveguide loss. We assume a coupler coupling efficiency of 90% at the half-harmonic signal frequency. We assumed realistic ranges of the expected second-harmonic generation efficiency and waveguide loss parameter and estimated the on-chip pump threshold power in the CW approximation. Our experimentally observed on-chip threshold corresponds to $\sim$ 50 mW of peak power. The theoretical estimate may be slightly lower than the experimental value owing to the following: incorrect assumption of the coupler response, neglecting the nonlinear loss at the pump wavelength due to the pump second harmonic generation, and the coupling of the pump power to higher-order modes which is not phase-matched and do not participate in the parametric oscillation.

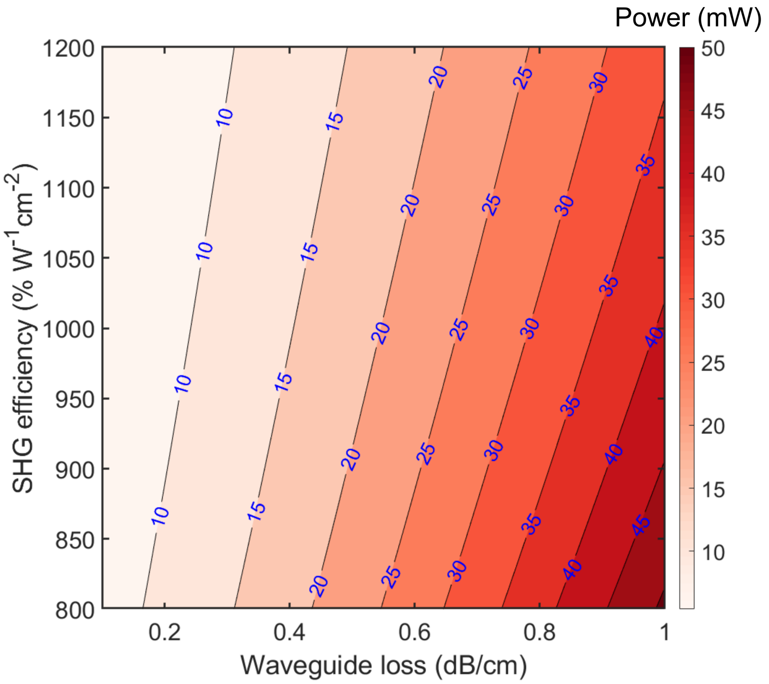

Supplementary Figure S2: Estimated threshold of the near-degenerate doubly resonant OPO (CW approximation) assuming a coupler coupling efficiency of 90% at the half-harmonic signal frequency.

2 Single-envelope simulation and visible frequency comb:

To capture the process of the generation of the second-harmonic and sum-frequency generation signals (responsible for the generation of the visible frequency comb), we resort to single nonlinear envelope simulation [2]. The numerically obtained results are shown in Fig. S3(a), which alludes to the existence of the visible frequency comb components. We have assumed the presence of non-idealities in the poling period in our simulation. We note that these second-harmonic and sum-frequency generation components are generated due to parasitic phase-matching owing to duty-cycle errors and/or higher-order phase-matching for the visible components.

In order to enhance the efficiency of the visible frequency comb generation process one can add an additional phase matching section at the output waveguide of the OPO. This would boost the conversion efficiency for the phase-matched component, and can also be designed to be broadband using chirped poling periods. Such a scenario where the efficiency of the SFG component between the pump and the signal is boosted is simulated in Fig. S3(b). Similarly, it can also be designed for the other components, namely the SFG of the idler and the pump, or the second harmonic frequency combs. We note that these visible frequency combs are single-pass due to the long-pass nature of the spectral response of the adiabatic couplers. The visible components inherit their wide tunability for their parent signal/idler frequency combs.

The fine tunability of the visible frequency comb (sum frequency generation between the signal and the pump) can also be performed using pump wavelength control as shown in Fig. S3(c).

The up-conversion resulting from the second-harmonic generation of the signal also leads to near-infrared frequency comb generation. The phase-matching curves are shown in Fig. S4(a). The coarse tuning and fine-tuning of the second-harmonic signal comb as obtained experimentally are shown in Fig. S4(b) and Fig. S4(c) respectively.

The up-conversion processes are not exactly quasi-phase-matched. Instead, the phase-matching curves (dotted lines) shown in Fig. 1 of the main text only denote energy conservation relation. The up-conversion processes still exist and are significant owing to the presence of random duty cycle errors [3, 4]. The periodicity of the poling can be controlled very accurately (sub 10 nm), but the duty cycle can be randomly varying. This causes a broad pedestal in the phase-matching that results in finite conversion efficiency for the up-conversion processes. This efficiency can be significant in the context of sync-pumped OPOs as pointed out in reference [4]. Figure S5(a) shows a second-harmonic microscopy poling image. The distribution of the random duty cycle is extracted in Fig. S5(b) from an ensemble of several poling images.

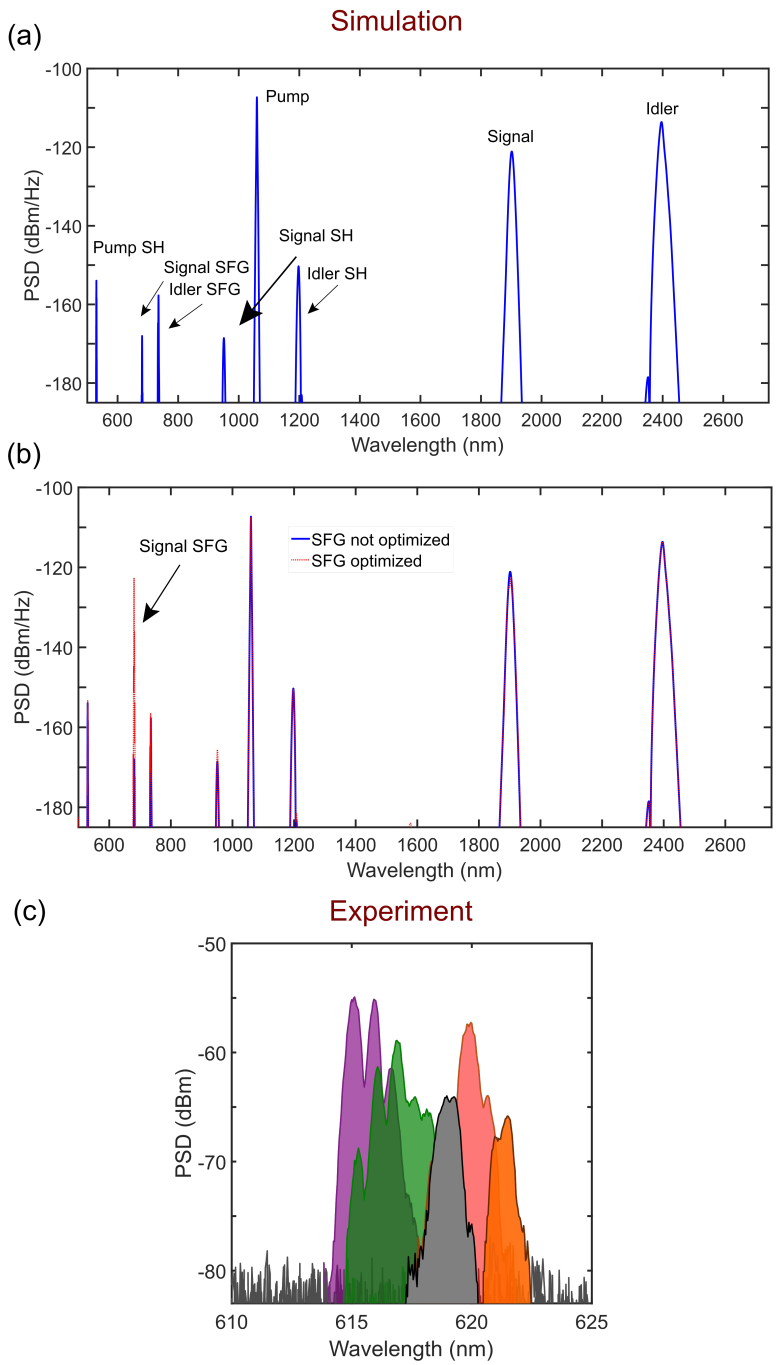

Supplementary Figure S3: **Visible frequency comb generation.** a), b), c) Experimentally obtained tunable visible frequency comb (sum frequency generation between the signal and the pump) via pump frequency tuning.

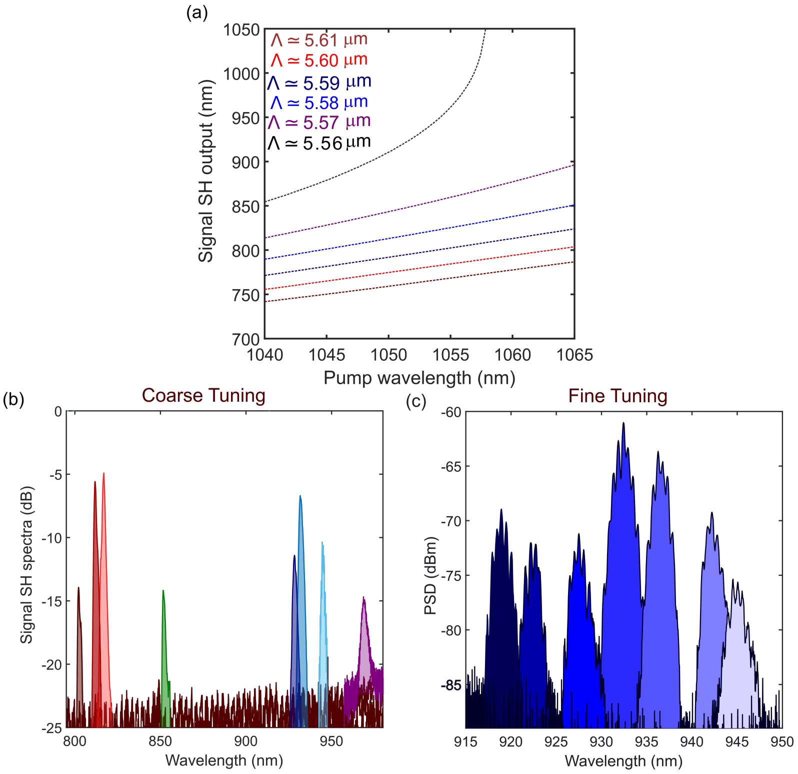

Supplementary Figure S4: **Second harmonic generation of the signal frequency comb.** a) Phase matching curves for different poling periods corresponding to the second-harmonic generation of the signal frequency comb b) Coarse tuning of the second harmonic of the signal frequency combs as obtained from the OPO chip, c) Fine tuning of the second harmonic of the signal frequency combs as obtained from a single OPO via pump wavelength tuning.

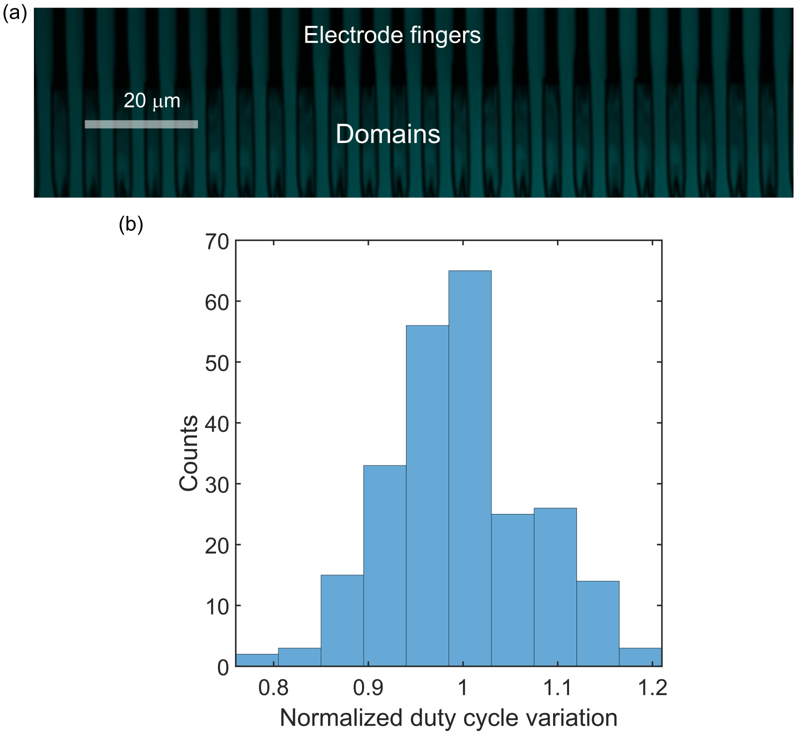

Supplementary Figure S5: **Random duty-cycle error in periodic poling.** a) Second-harmonic microscopy image of a periodically poled region b) Distribution of poling duty cycle extracted from an ensemble of several poling images.

3 Signal/Idler conversion efficiency:

We plot the signal power as a function of the pump power as shown in Fig. S6(a). The OPO conversion efficiency is a function of the escape efficiency, number of times above threshold operation, etc.. [5]. OPOs operating at far non-degeneracy will have significantly different conversion efficiency corresponding to the signal and the idler owing to the coupler response which enforces a strongly resonant idler while the signal remains weakly resonant. The ratio of the signal to idler power varies as shown in Fig. S6(b).

The conversion efficiency estimations and the fit to the simplified model neglect the contributions of the upconversion processes, namely the SHG and SFG processes.

The escape efficiency is determined by the OPO-cavity output coupling, which is given by the frequency response of our adiabatic coupler. The schematic of the geometry of our coupler is shown in Fig. S6(c). The geometrical parameters are mentioned in the caption of Figure S6. The simulated performance from the coupler is shown in Fig. S6(d).

The coupler response is also characterized experimentally by illuminating with a super-continuum source. The results are overlaid in Fig. S6(e).

We measure an off-chip mid-infrared power of $\sim$ 300 nW. The spectrum is shown in Fig. S7. The corresponding on-chip average power is $\sim$ 3 μW. The estimated pulse width for the idler is $\sim$ 500 fs (transform-limited). This indicates a peak power of $\sim$ of 25 mW. The power per comb line is $\sim$ 5 μW. Note that we have multiplied the power levels with the quasi-pulse duty cycle of 100.

The threshold of the extreme mid-infrared OPO is estimated to be approximately 10 times that of the near-degenerate OPO (possessing a threshold of $\sim$ 1 mW of average power).

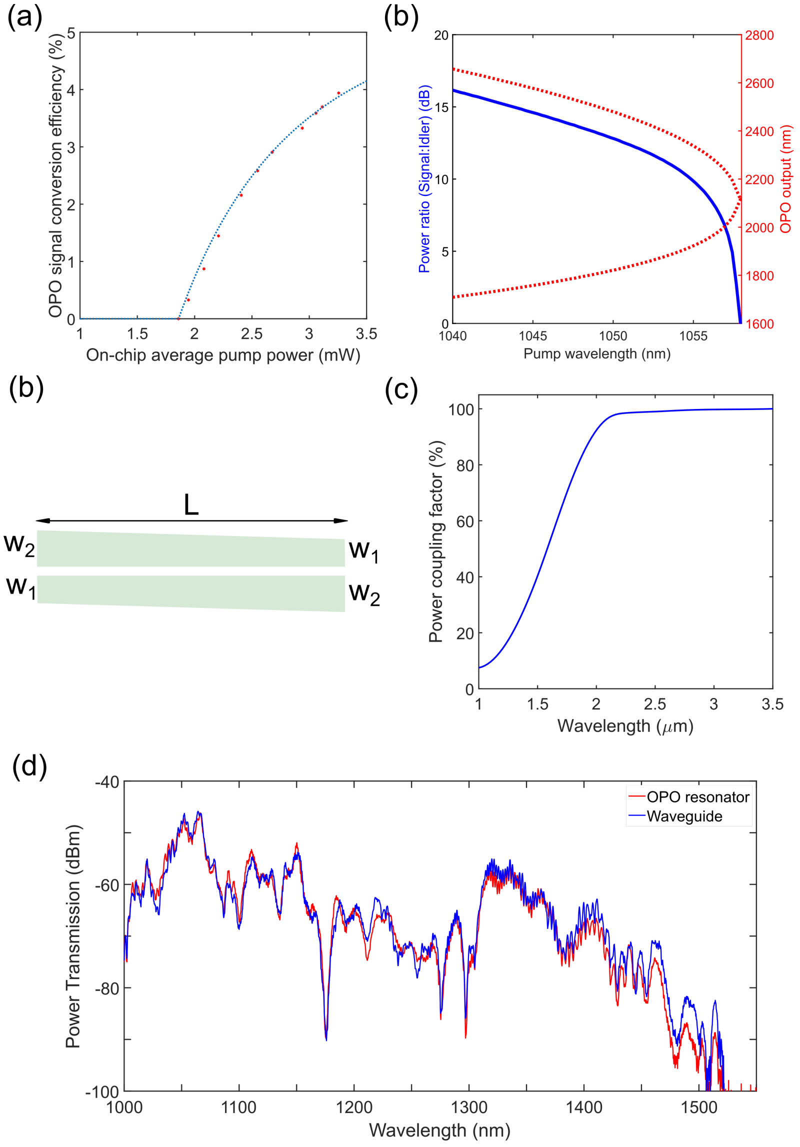

Supplementary Figure S6: **Coupler response and OPO conversion efficiency.** a) Signal conversion efficiency as a function of on-chip pump power. The experimental data fit well with the analytically expected scaling of $\frac{\sqrt{N}-1}{N}$, where N, is the number of times above threshold operation. b) Schematic of the adiabatic coupler with the following parameters: $w_1 = 2.3\mu$m, $w_2 = 2.5\mu$m, gap $= 1\mu$m, and length $L = 500\mu$m. c) Simulated Response of the adiabatic coupler, showing that it transmits most of the pump wavelengths (around $1\mu m$) while coupling the majority of the power contained in the longer wavelengths ($>2\mu m$) d) Measured transmission of a supercontinuum source through the OPO resonators containing the couplers (red curve). The response for the case of transmission through a straight waveguide is shown for reference (blue curve). It can be seen that at longer wavelengths the transmission through the OPO resonators dips on account of the coupling response of the couplers.

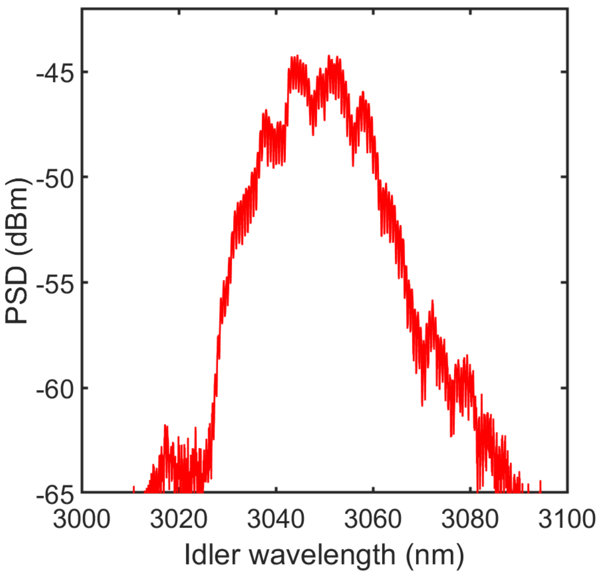

Supplementary Figure S7: Mid-infrared frequency comb (Idler frequency comb) spectrum obtained using an optical spectrum analyzer that is used for the calculation of power per comb line. Optical spectrum analyzer settings: Resolution bandwidth 1 nm, sampling interval 0.05 nm, and frequency comb repetition rate $\sim$ 19 GHz.

4 Pump preparation/ Electro-optic frequency comb generation:

The OPO is pumped by an electro-optic frequency comb whose repetition rate is tuned close to the cavity FSR. The pump pulse width is approximately 1 ps long, and based on the available electronics in our current version the repetition rate can be tuned from 5 GHz to 20 GHz (the upper limit is dictated by the bandwidth of the RF amplifiers). The electro-optic frequency comb generation scheme closely follows the approach demonstrated in [6, 7]. The center frequency can be tuned from 1040 nm to 1065 nm (the upper limit is determined by the operating range of the waveshaper, while the lower limit is chosen to ensure the safe operation of the YDFA).

The schematic of the pump preparation setup is shown in Fig. S8(a). The output of the CW laser is modulated by a series of modulators. The modulators are driven by an RF signal generator followed by an RF amplifier. The Intensity modulator (IM) bias is chosen such that pulses can be carved out from the continuous wave. At this stage (Stage 1) the time domain output resembles the simulated waveform shown in Fig. S8(b). Next, a cascade of 3 Phase modulators (PM) enables the addition of spectral sidebands which are separated by the repetition rate. The Phase modulators are driven in sync by adjusting the electronic delay lines. At this stage (Stage 2) the spectrum will be similar to the one shown in Fig. S8(c). The resultant signal is amplified with the help of a semiconductor optical amplifier (SOA) and then sent to a waveshaper. The programmable waveshaper allows the compression of the pulses by de-chirping the input temporal waveform through the application of suitable dispersion. At this stage (Stage 3), the time domain waveform will look like the one shown in Fig. S8(d), where both the compressed pulses as well as the pre-compressed chirped pulses are shown. Finally, the electro-optic frequency comb is characterized in the frequency domain using an optical spectrum analyzer (OSA), and in the time domain using an intensity auto-correlator.

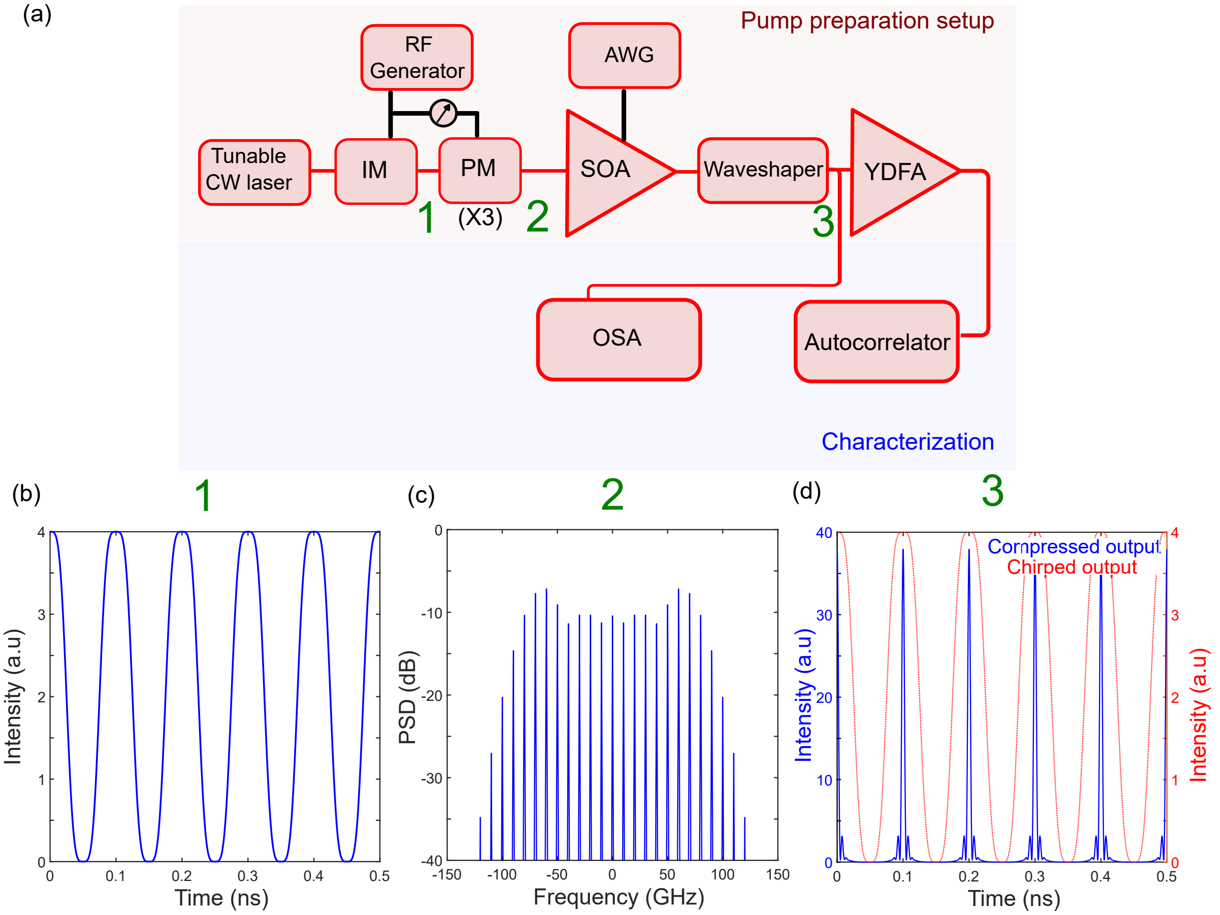

Supplementary Figure S8: Pump preparation setup. a) Schematic of the setup for electro-optic comb generation that is used for the sync-pumping of the OPO. Arbitrary Waveform Generator (AWG), Semiconductor Optical Amplifier (SOA), Intensity Modulator (IM), Phase Modulator (PM), Ytterbium Doped Fiber Amplifier (YDFA), Optical Spectrum Analyzer (OSA). b,c,d) Simulated waveforms in the time and frequency domain at different stages of the pump preparation setup.

5 Quasi-sync pumping:

The threshold of the far non-degenerate OPOs is higher owing to a combination of multiple reasons. The adiabatic coupler is not tailor-designed for each OPO, instead, a uniform coupler has been implemented in this first-generation chip design. As a result, the far non-degenerate operation of the OPOs leads to signals experiencing higher round-trip losses (due to progressively larger out-coupling for smaller wavelengths). Moreover, the effective nonlinear coefficient which takes into consideration the effective area of the modes, and the field overlap between the pump, signal, and idler modes also degrades.

The higher threshold requirement demands more pump power which is currently on the higher side due to the rather high input coupling loss/ insertion loss (approximately between 10 to 12 dB). There have been several proposals and demonstrations to bring this number down to a few dBs [8, 9]. In the

scenario of the availability of low insertion loss, the required external pump power can be dramatically reduced by approximately 10 dB. Under these circumstances, the threshold requirement for far non-degenerate operations can be easily accessible even with sub-optimum design.

However, in our present implementation, we, unfortunately, do suffer from excess insertion loss, which results in the required off-chip average power exceeding 60 mW. At these power levels, we are prone to burning/ damaging the connectors and affecting the YDFA in the presence of undesired back-reflected power. To ensure safe operation we resort to quasi-sync pumping, whereby the average power is reduced by pulsing the pump. This can be achieved by driving the semiconductor optical amplifier (SOA) using an arbitrary waveform generator (AWG) leading to microsecond scale pulses at a repetition rate varying from 1 to 20 KHz (Duty cycle of 1000 to 50). The schematic is shown in Fig. S9(a), which is the same as the pump preparation setup with the addition of an AWG-driven SOA. The time domain traces captured using a slow detector for the pump and signal pulses are shown in Fig. S9(b). We note that the slow detector could not resolve the individual picosecond scale pulses within each quasi-pulse. In fact, there are of the order of thousands pulses within each quasi-pulse.

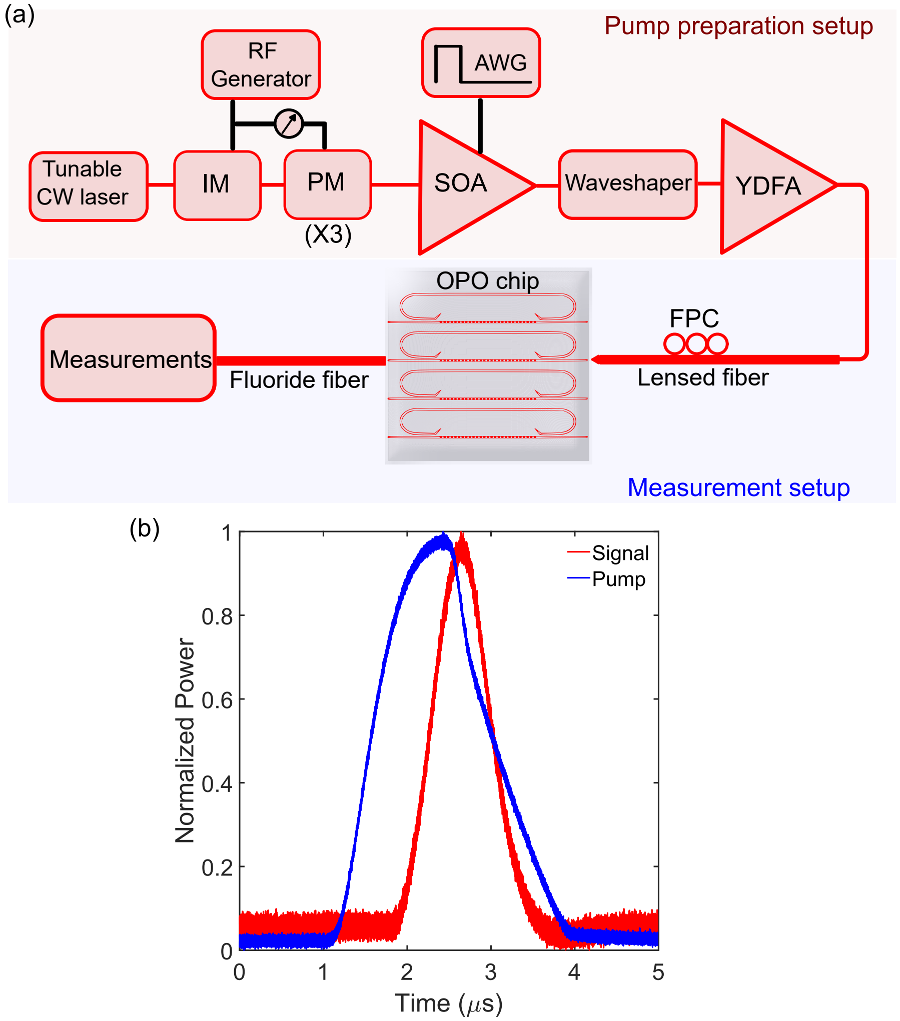

Supplementary Figure S9: Quasi-sync pumping arrangement. a) Schematic of the setup for electro-optic comb generation that is used for the sync-pumping of the OPO in the quasi mode of operation. Arbitrary Waveform Generator (AWG), Semiconductor Optical Amplifier (SOA), Intensity Modulator (IM), Phase Modulator (PM), Ytterbium Doped Fiber Amplifier (YDFA), Optical Spectrum Analyzer (OSA). b) Measured time domain trace of the quasi pulses for the pump (blue) and the signal (red) quasi pulses using a slow detector.

6 Estimating the cavity free spectral range:

Estimating the free spectral range of the cavity (FSR) is central to determining the repetition rate of the synchronously pumped OPO. This is absolutely necessary since the sync-pump (EO comb) cannot be tuned continuously to search for the right FSR. Each setting of the EO comb requires a specific combination of the electronic phase delay line parameters and the waveshaper dispersion parameter, adjusting which is an arduous task. The design of our OPO precludes the use of a tunable CW source around 1 μm to scan through multiple cavity resonances. The situation is exacerbated in the absence of

a high-power tunable CW source of around 2 μm at our disposal. Under these circumstances, we estimate the cavity FSR using a measurement setup as shown in Fig. S10.

In this approach, we have to operate the OPO in CW mode. We apply a variable modulation on top of the CW using an intensity modulator (IM). The frequency of modulation is varied using an arbitrary waveform generator. The output of the OPO will be maximized in the vicinity of the correct cavity FSR. This setup unlike the EO comb can be continuously tuned.

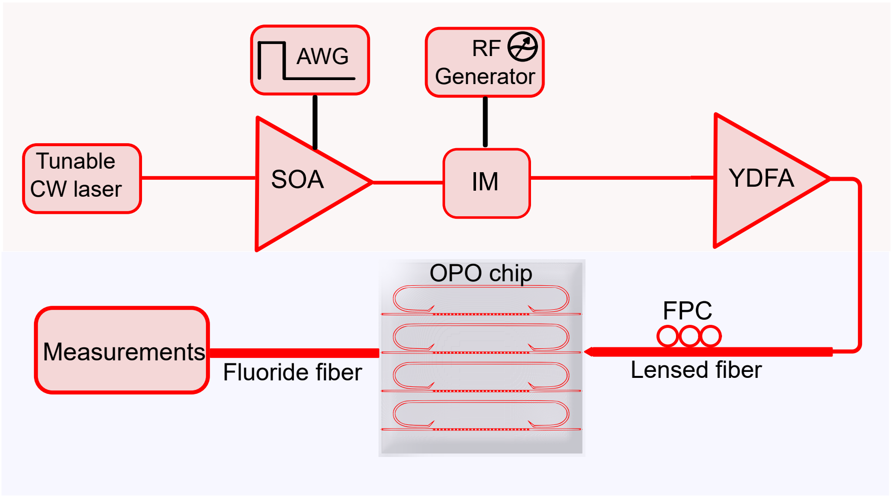

Supplementary Figure S10: **Schematic of the setup for estimating cavity FSR**. Arbitrary Waveform Generator (AWG), Semiconductor Optical Amplifier (SOA), Intensity Modulator (IM), Ytterbium Doped Fiber Amplifier (YDFA).

For near-degenerate OPOs, the group index of the signal and the idler will be similar. However, for far non-degenerate OPO operation the group index for the signal and idler can be quite dissimilar resulting in the signal and idler waves experiencing different cold-cavity FSR. Owing to the transmission nature of the adiabatic coupler the idler wave will be more resonant than the signal. Thus the lowest threshold is expected when the pump repetition rate matches the idler FSR. As we detune the repetition rate from being exactly matched with the idler FSR and instead try to match it with that of the signal the threshold will increase. This fact is represented using numerical simulation by varying the repetition rate as shown in Fig. S11.

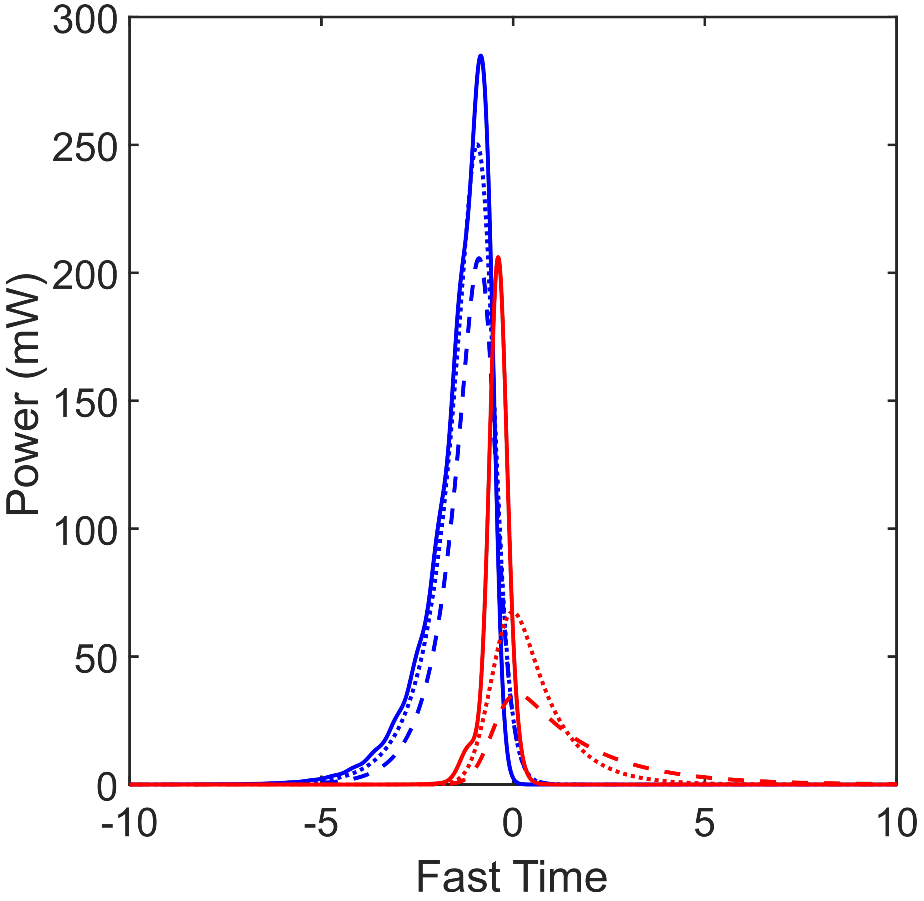

Supplementary Figure S11: **Repetition-rate dependence for far non-degenerate OPO operation.** The blue and red curves denote signal and idlers respectively obtained by numerical simulation. The solid line corresponds to the case where the pump repetition rate is matched with the cavity FSR corresponding to the idler. The other two cases (i.e. the dashed and dotted curves) represent scenarios where the repetition rate is not exactly matched for the idler but instead approaches the FSR of the signal. The dashed line represents the scenario where the repetition rate is closer to the signal FSR as compared to the dotted one.

7 Passive stability and frequency comb locking:

A fully-stabilized frequency comb can be used as a frequency reference. This translates to locking both the repetition rate and the carrier-envelope phase offset. In the case of synchronously pumped OPO, the repetition rate of the output is given by the pump repetition rate, which in our case is the applied modulation from the RF generator in the electro-optic comb generation process.

The other degree of freedom is the carrier-envelope offset frequency (f_{CEO}) of the OPO frequency comb. OPOs operating at degeneracy have the f_{CEO} locked to the pump frequency comb and assume the value of either $\frac{f_{CEO,p}}{2}$ or $\frac{f_{CEO,p}}{2} + \frac{f_{REP}}{2}$ [10]. In this case, locking the pump f_{CEO} automatically guarantees the f_{CEO} locking for the sub-harmonic component. This is the divide-by-2 instance. Another special scenario arises in the divide-by-3 case, where the relation between the frequencies is the

following: pump (ω_p), signal ($\frac{\omega_p}{3}$), and the idler ($\frac{2\omega_p}{3}$). In the presence of a second-harmonic coupling between the idler and the signal, this would also result in a locked signal and idler OPO frequency comb, provided the pump f_{CEO} is locked [11, 12]. However, these are special cases of self phase locking and will not be applicable in general.

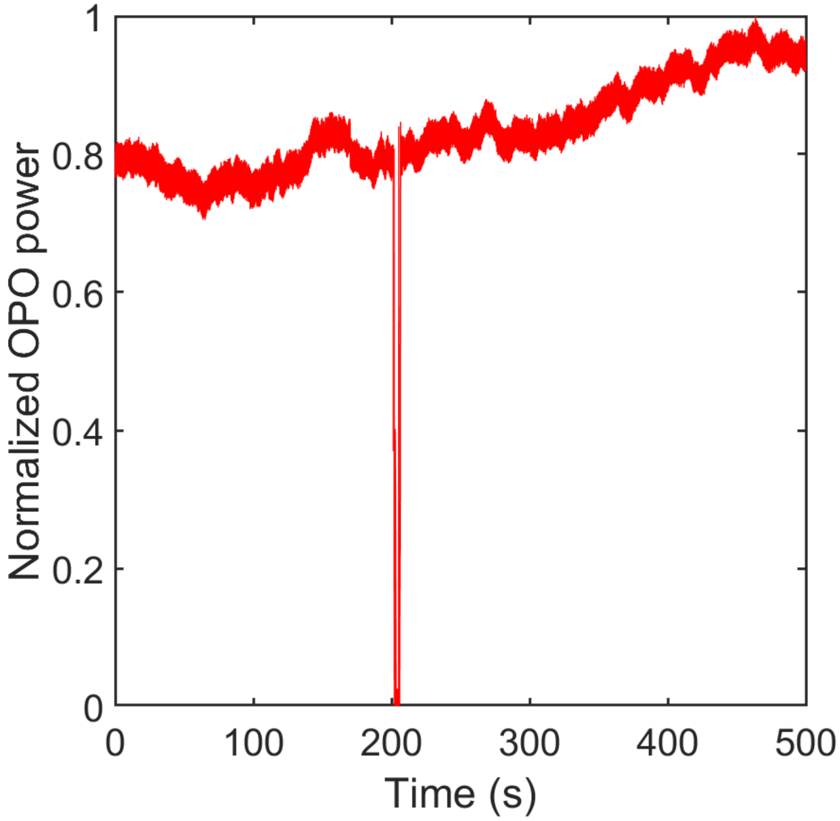

Supplementary Figure S12: Passive stability of the OPO output power as a function of time without any feedback locking. The OPO power is normalized with respect to the minimum and maximum power recorded over the interval of time. The OPO was momentarily disrupted by blocking the pump and again restored near the 200s time stamp as evident from the power drop and recovery. The OPO power remains fairly stable over the entire duration even without active feedback.

A more general locking scheme will involve locking two degrees of freedom. This can be for example: locking the f_{CEO} of the near-infrared pump frequency comb and the f_{CEO} of the near-infrared signal frequency comb. This will ensure that the idler frequency comb is locked. If we don't need an absolute frequency reference but are satisfied with a stable frequency comb source (f_{CEO} drift free), then we can lock the f_{CEO} of the pump frequency comb and the cavity detuning. This arises because of the fixed relationship between the cavity detuning and the f_{CEO} variation in the case of doubly-resonant non-degenerate synchronously pumped OPOs [13].

The visible component of the frequency comb can also be used for comb stabilization purposes. In this case, the visible component can be stabilized to an atomic transition or so.

A device capable of producing a stabilized frequency comb in a general scenario will require two tuning knobs. This can be the current control of the CW laser source whereby one can leverage the current driven frequency drift property. The other knob could be an intra-cavity modulator (electro-optic for fast feedback, and/or thermo-optic for slow feedback) which would be responsible for the cavity detuning locking purpose.

In our present device, we do not have an integrated modulator in place. The subject of investigating frequency and power stability will be a subject of future investigation. Here, we show experimental data for the power stability in a passive scenario, i.e. without engaging any feedback control (Fig. S12). In the case, when the OPO is operating way from degeneracy, the OPO output is seen to be fairly stable over several minutes of operation. It gets affected in the long time scale due to a multitude of factors namely the coupling variation etc.., which needs further investigation.

8 Mid-infrared absorption and it's near-infrared counterpart:

The generated mid-infrared frequency combs from our OPO chip extend all the way up to 3.3 μm (detected with the OSAs available in our lab) and beyond (inferred from the associated signal spectrum in the near-infrared). We already see absorption features in our mid-infrared idler spectrum (see Fig. S13). This is owing to several absorption lines in the frequency range of interest experienced by various organic molecules. We have not yet characterized the origin of the molecules/ samples responsible for the absorption, which will be a subject matter for the future investigation leading to on-chip spectroscopic applications. This can lead to a powerful spectroscopic platform, thanks to the possibility of integrating the sample in the intra-cavity of the OPO.

The intra-cavity absorption not only allows to enhance the sample interaction with the mid-infrared light, but it can also be extremely useful owing to the inherent spectral correlations between the signal and idler. The idler absorption feature will be reflected onto the signal spectrum, thereby paving the way for mid-infrared spectroscopy via the detection of near-infrared photons.

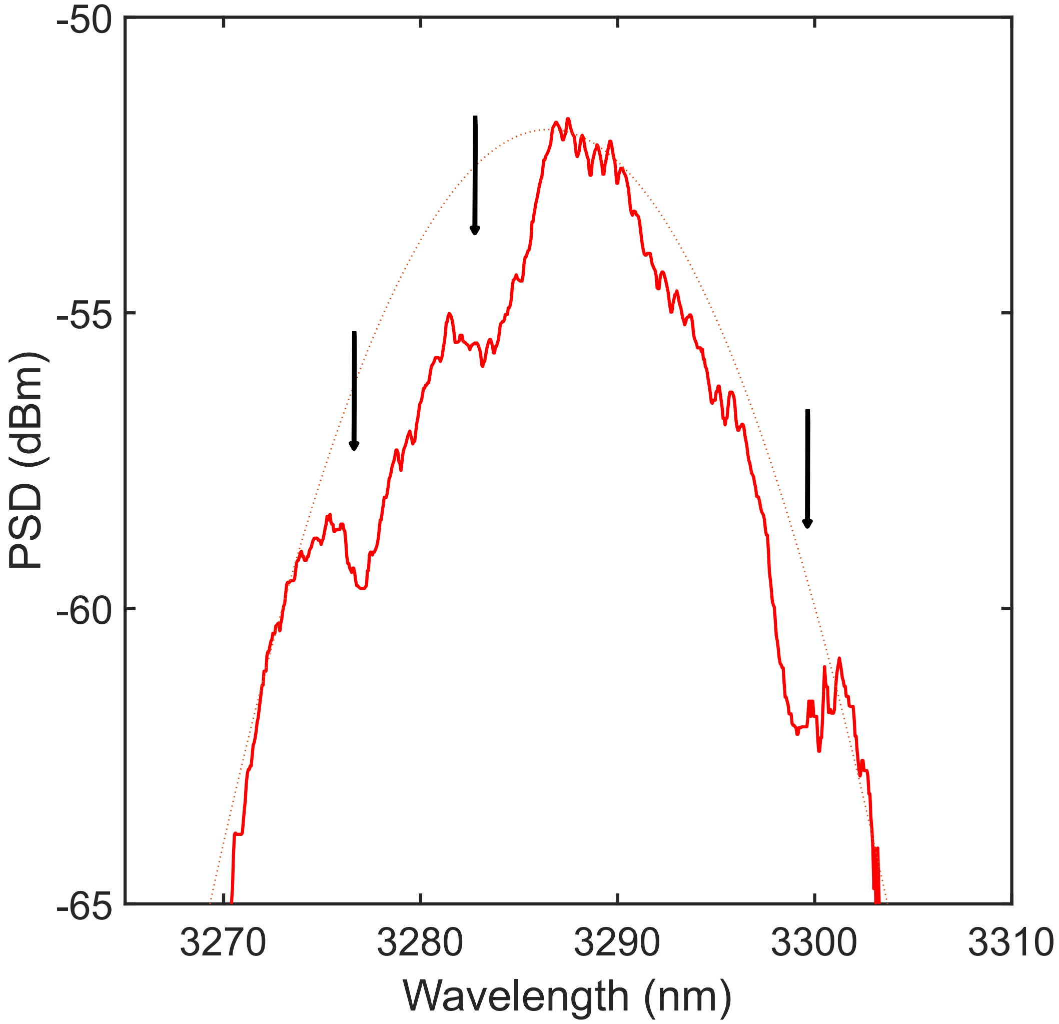

Supplementary Figure S13: Idler spectrum in the mid-infrared containing absorption features. The dotted line is a Gaussian envelope fit. The arrows indicate the existence of absorption features as evident from the deviation of the spectral envelope from the smooth Gaussian envelope.

9 Spectral broadening/ Pulse compression in the degenerate OPO:

The measured pump pulse width (assuming a Gaussian pulse as extracted from the intensity auto-correlation trace) is ~ 1 ps. The estimated transform-limited pulse width for the OPO operating at degeneracy is 380 fs. This translates to a spectral broadening/ pulse compression factor of ~ 2.6. The experimental spectrum of both the pump and signal are translated in frequency and overlaid on top of each other as shown in Fig. S14(a). The numerical simulation results obtained from a dual-envelope equation simulation are shown in Fig. S14(b-e), which closely agrees with the measured data. By proper

dispersion engineering (group velocity dispersion and group velocity mismatch) [14], it is possible to generate OPO frequency comb with broad instantaneous bandwidth, leading to few optical cycle pulses.

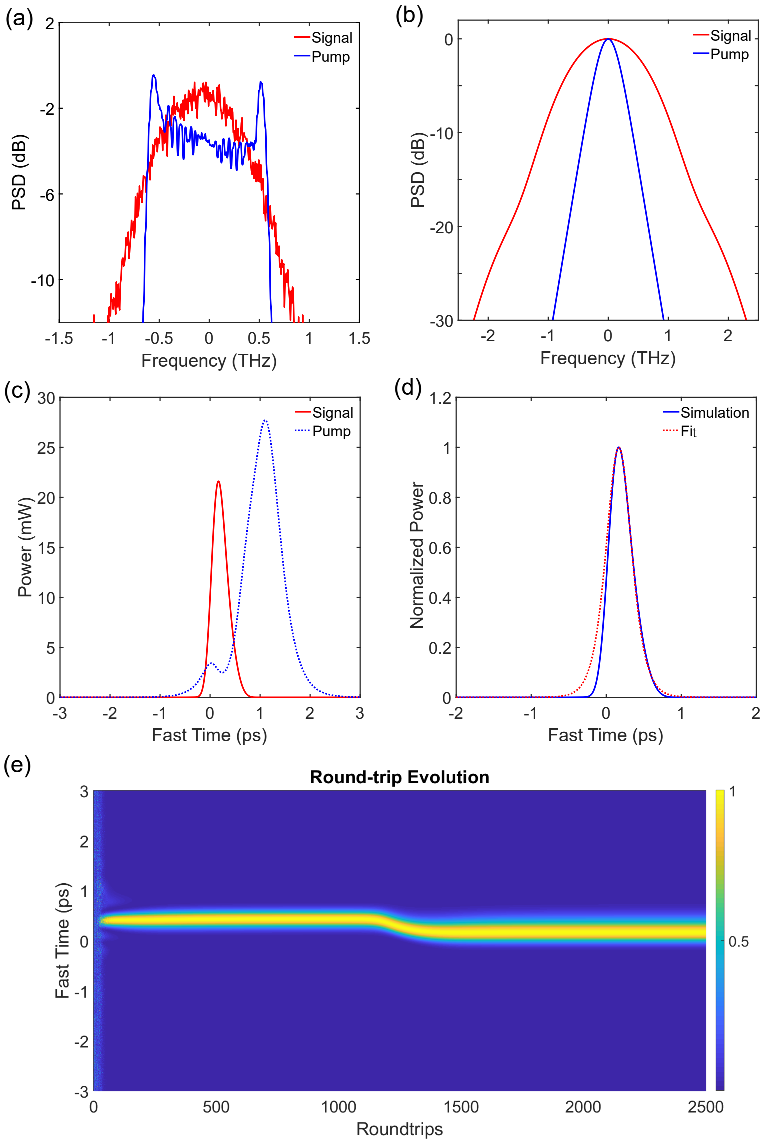

Supplementary Figure S14: **Spectral broadening and associated pulse compression in the degenerate regime of OPO operation.** a) Experimentally obtained spectrum plotted in the frequency axis after spectral translation showing the effect of spectral broadening in the signal compared to the pump, b) Spectrum obtained from the numerical simulation showing the spectral broadening effect (assuming a sech-shaped pump pulse), c) Pump and signal pulses in the time domain as obtained from the numerical simulation, d) The numerically obtained signal pulse when fitted with a sech shaped pulse denotes a pulse compression by a factor of approximately 2, e) The normalized roundtrip evolution of the signal pulses that are initiated from the vacuum field till it reaches the steady state.

10 Carrier-Envelope offset frequency of the OPO frequency comb in the quasi-mode of operation:

When the OPO is operated in the quasi-sync pumped mode, the intensity of the pump as well as the generated signal/idler pulses change over the course of the duration of the quasi pulse as shown in Fig. S9(b). This would lead to an intensity-dependent carrier-envelope offset frequency shift for the OPO frequency comb. This can arise due to several intensity-dependent effects like thermo-optic effect induced detuning change, or the intrinsic Kerr nonlinearity-induced detuning variation within the duration of the quasi pulse [15]. We measure this frequency drift for a non-degenerate OPO whose signal lies around 1550 nm (on account of the availability of a CW laser that acts as a local oscillator and fast photo-detector). The frequency drift of the OPO frequency comb that is reflected in the beat note is shown in Fig. S15(c). We confirmed that it doesn't arise from the pump frequency drift by observing the beat note of the pump frequency comb with another CW local oscillator around 1060 nm as shown in Fig. S15(b). The schematic of the beat-note detection setup is shown in Fig. S15(a). This intensity-dependent effect can also be used to stabilize the OPO frequency comb, whereby the pump power can be used as a feedback knob.

We expect that the frequency drift will not arise in the case of a complete sync-pumped mode of operation, since the OPO operates in a steady state, with intensity fluctuations only happening in the long time scale. We couldn't verify it due to the absence of a tunable local oscillator around 2.1 μm, where the OPO operates in the near-degenerate regime and possess a lower threshold which can be surpassed in the complete sync-pumped mode of operation. We also expect in the degenerate mode of operation this frequency drift will be absent even in the quasi-sync pumped case, owing to the sub-harmonic frequency locking between the pump and the signal.

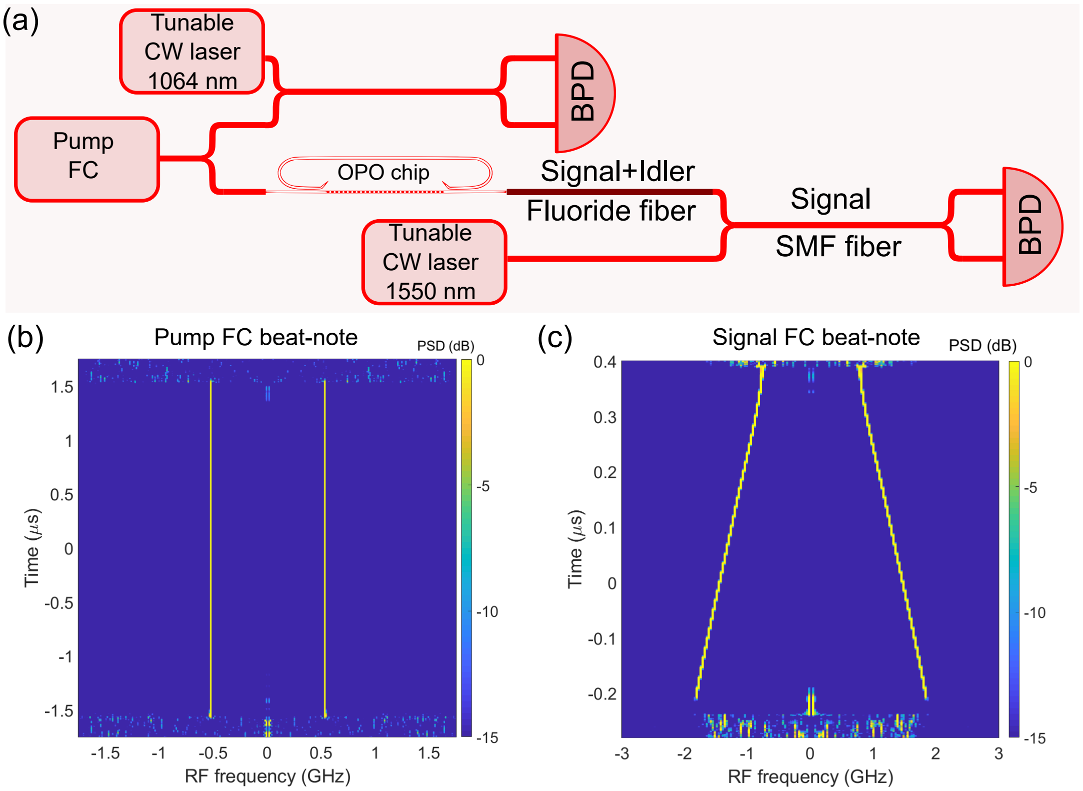

Supplementary Figure S15: **OPO frequency comb beat-note in the presence of quasi-sync pumping**. a) Schematic for the setup used to measure the beat-note for the pump and signal frequency comb. Abbreviations used are Balanced Photo-diode (BPD), and Frequency Comb (FC). b) beat-note evolution over a single quasi pulse in the pump frequency comb showing the absence of frequency drift, c) beat-note evolution over a single quasi pulse in the signal frequency comb showing the presence of frequency drift owing to the detuning variation arising from several nonlinear effects (Kerr-effect/ thermo-optic/ photo-refractive effect). This drift is however deterministic as the same pattern evolves over consecutive quasi-pulses.

11 Coherence verification using field cross-correlation technique:

In order to evaluate the coherence of the spectrum, we perform a linear field cross-correlation (FCCR) of the output signal light, where each OPO pulse is interfered with another pulse delayed by 10 roundtrips. This can be thought of being a modified FTIR measurement, where instead of performing auto-correlation we are executing cross-correlation. The schematic of the setup used for this purpose is shown in Fig. S16(a). The delay line corresponds to a delay of 10 OPO pulses, and thus the coherence property evaluation is limited by the applied delay duration. The scanning stage nonlinearity is corrected using a reference HeNe laser beam. This is important to match the optical spectrum obtained through the optical spectrum analyzer and which is calculated by performing the Fourier transform of the FCCR trace.

We also detect a sharp RF beat frequency corresponding to the applied repetition rate of the sync-pumped OPO (Fig. S16(b)). The signal is obtained by measuring the OPO output pulses using a fast photo-detector. The pump is rejected using a wavelength de-multiplexer.

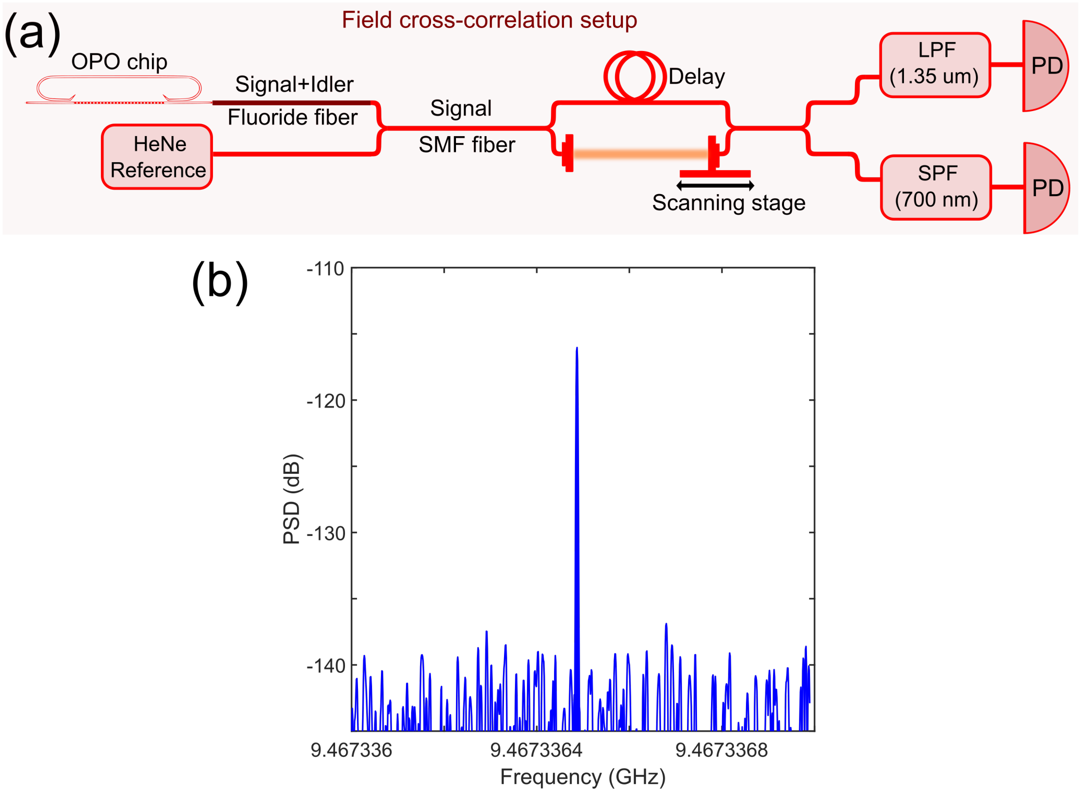

Supplementary Figure S16: Frequency comb coherence. a) Schematic of the setup used to verify the coherence of the OPO output b) Measured RF beat-note frequency corresponding to the applied repetition rate of the sync-pumped OPO.

12 Full system integration and a universal frequency comb source

We envision a complete integrated solution for frequency comb generation based on lithium niobate nanophotonics in conjunction with a laser chip. With several design enhancements, it is possible to lower the threshold for frequency comb generation substantially which can allow the pumping with commercially available DFB laser chips. Alternatively, an integrated external cavity along with a semiconductor gain chip can also be deployed for this purpose [16]. The other crucial building blocks are: a) near-IR picosecond pump pulse generation [17, 18], b) Mach Zehnder interferometer mesh for routing the pump light to the desired OPO [19], c) an array of OPOs, and d) periodically poled lithium niobate waveguides supporting ultra-low power supercontinuum generation for f-2f based frequency

comb stabilization [20, 21]. Our present work focuses on part c, while the rest has already been demonstrated in lithium-niobate nanophotonics.

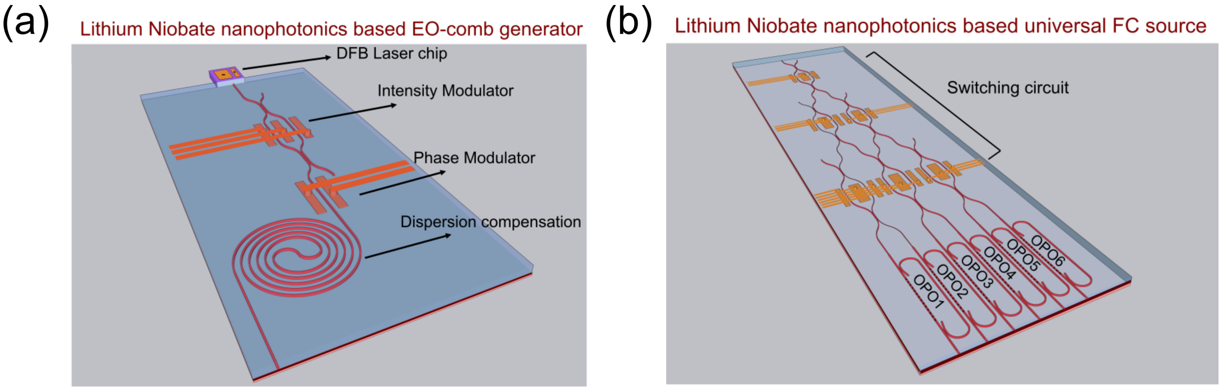

Supplementary Figure S17: Artistic illustration of the proposed universal frequency comb source. a) Illustration of a lithium niobate nanophotonics-based near-IR picosecond pump source consisting of a cascade of intensity and phase modulators followed by a de-chirping spiral waveguide b) Illustration of a lithium niobate OPO chip consisting of an array of OPOs dedicated to cover different spectral regions which can be programmatically pumped with the help of the MZI routing circuit preceding it.

13 Temperature tuning of the phase-matching curves

The fine-tuning of the quasi-phase-matching (QPM) in the present work has been performed by tuning the pump wavelength. The same can be achieved with the help of temperature tuning while keeping the pump wavelength fixed. Figure S18 shows the phase-matching curves as a function of temperature which are calculated by evaluating the effective index of the waveguides taking into consideration the temperature-dependent Sellmeier equation [22]. Temperature tuning can either be attained globally by placing the chip on top of a TEC heater element, or locally by implementing resistive heater elements close to the periodically poled regions. We note that the expected tuning curves (obtained from simulations) are more tunable than what is observed in experiments. We anticipate that it may be attributed to the presence of thermal resistance between the heater element and the nanophotonic chip, and/or the mismatch in the thermal expansion coefficients between the insulator layer and the thin-film lithium niobate layer. This a subject of ongoing investigation.

We observed the tuning of the OPO spectrum as the temperature of the whole chip (global temperature variation) is varied. While we are confident about the temperature tuning of phase matching, systematic measurement of OPO output as a function of temperature is challenging with our current experimental setup. This is mainly because when we resort to changing the temperature of the chip, not only is the phase-matching in the periodically poled waveguide altered, but the ring cavity also experiences modification in its group index which in turn alters the FSR of the cavity. It is a cumbersome process to modify the EO comb pump for every setting of the temperature change which is required for a systematic study. Our EO comb comprises of a cascade of three phase modulators which are driven by amplified RF signal oscillating at the FSR frequency. This set of 3 amplifiers are preceded by corresponding phase delay units that are required to ensure all the phase modulators are driven in-phase. Every different setting of FSR enforces a different setting for these phase delay units and also a unique setting for the waveshaper. This is the reason we avoided the systematic study of temperature variations.

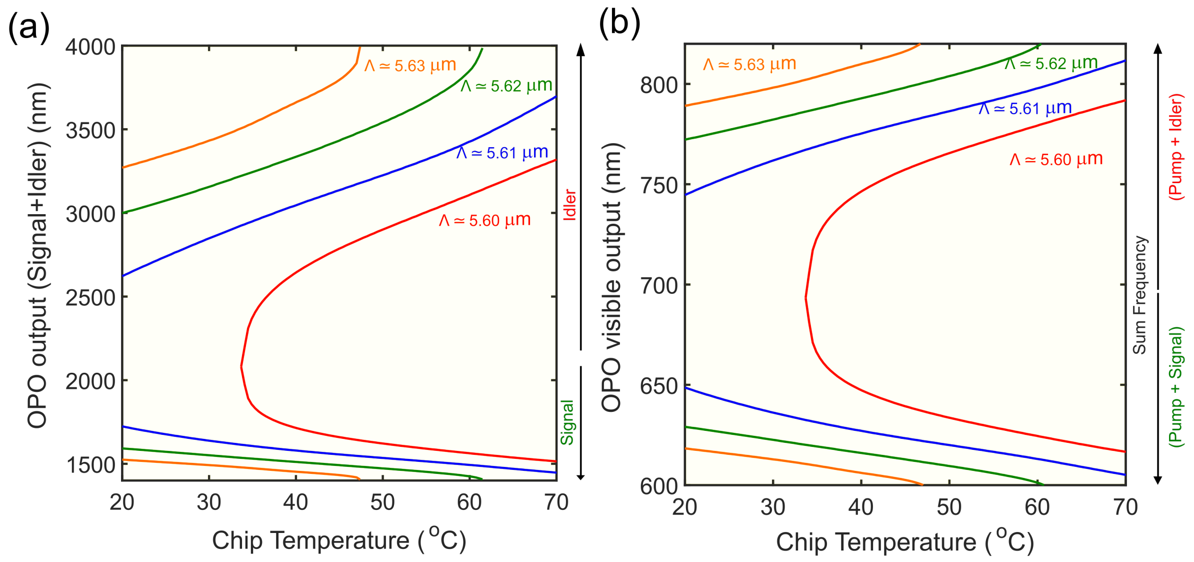

Supplementary Figure S18: Temperature tuning of the quasi-phase-matching obtained from simulation for the a) signal and the idler waves and b) their corresponding up-conversion with the pump leading to visible frequency combs. The pump wavelength is kept fixed at 1060 nm.

Below we present the effect of temperature variation on the phase-matching in periodically poled thin-film nanophotonic devices. Although these results showing the agreement between the simulation and experiment pertaining to temperature tuning hasn't been published before, the results are related to our previous work [23]. The device under consideration is an optical parametric amplifier, which is essentially a single-pass device, and hence the complexity of a global temperature change affecting the cavity FSR is absent in this case. Thus we can decouple the temperature effect on the phase-matching. Figure S19 shows how the optical parametric generation spectrum is affected by temperature variation. Results obtained by numerical simulation agree with the experimentally obtained data [23].

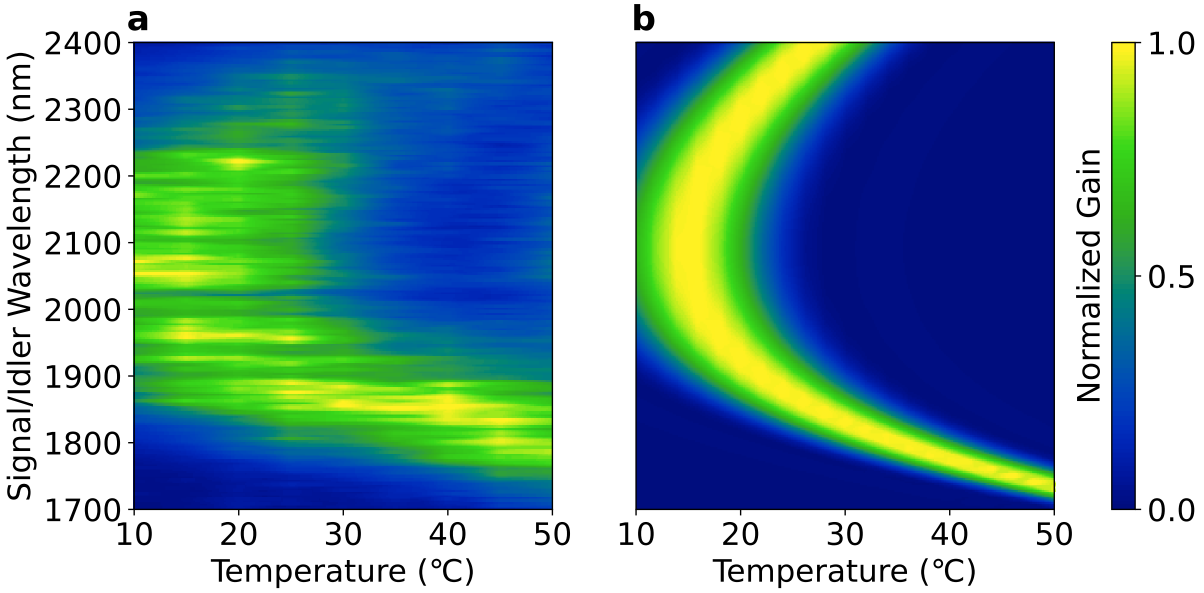

Supplementary Figure S19: Tuning of the OPG spectrum by varying the temperature of the chip as obtained a) experimentally and corroborated by numerical simulations in b). The data pertains to the device presented in [23].

14 Pump wavelength tuning in doubly-resonant OPO

For fine-tuning the frequency comb, we performed pump wavelength tuning. The tuning curve for a doubly-resonant OPO is complicated although deterministic and is characterized by peaks with distinct spectral features [24, 25]. Although locally the spectral tuning is complex, globally it follows the phase-matching curves. The tunability obtained by analyzing the phase matching curves can be expressed as:

$$\frac{\partial \omega_s}{\partial \omega_p} = \frac{n_{g,p} - n_{g,i}}{n_{g,s} - n_{g,i}} \tag{S.3a}$$

$$\frac{\partial \omega_i}{\partial \omega_p} = \frac{n_{g,s} - n_{g,p}}{n_{g,s} - n_{g,i}} \tag{S.3b}$$

where $\frac{\partial \omega_s}{\partial \omega_p}$, and $\frac{\partial \omega_i}{\partial \omega_p}$ represents the tuning slope of the signal and the idler with respect to the pump. In deriving these expressions we assumed small deviations. $n_{g,p}, n_{g,s}, n_{g,i}$ denotes the group index at the pump, signal, and idler frequencies respectively. For operations near degeneracy, the denominator can be quite small, thereby leading to a large tuning slope. The opposite behavior follows for far non-degenerate operations.

Some more fine-tuning spectra obtained experimentally in addition to the results shown in Fig. 3(c) of the main text are shown in Fig. S20 and Fig. S21.

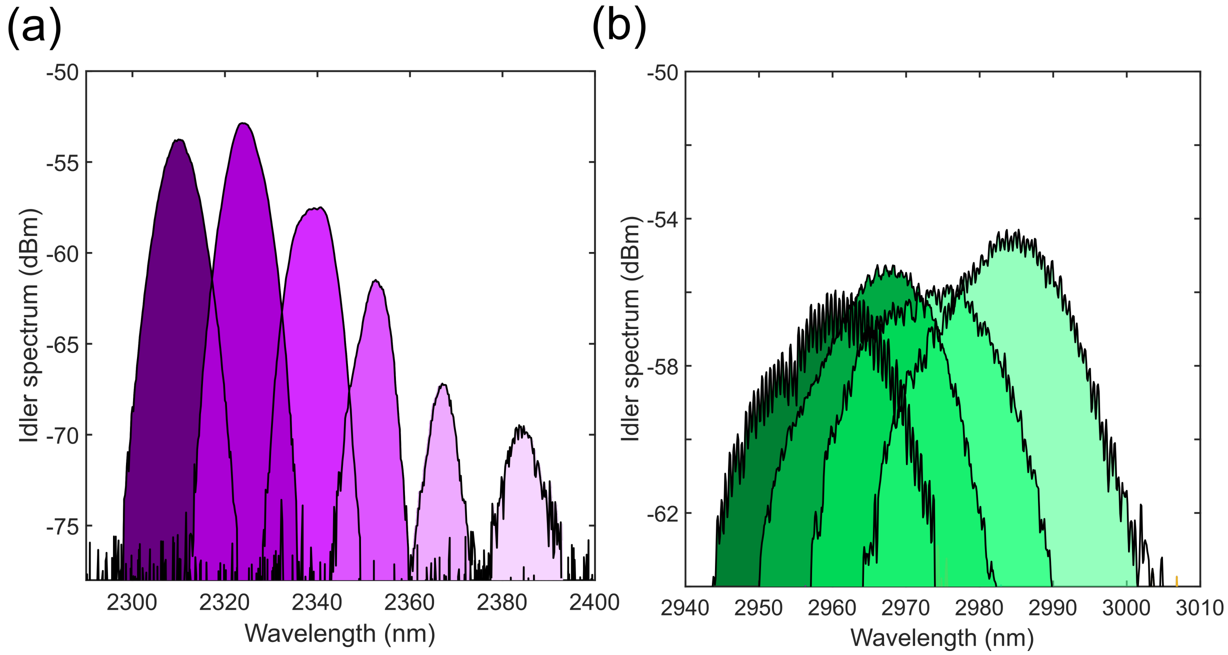

Supplementary Figure S20: Tuning of the idler frequency comb via changing the pump wavelength. a) and b) correspond to spectra obtained from OPOs with distinct poling periods.

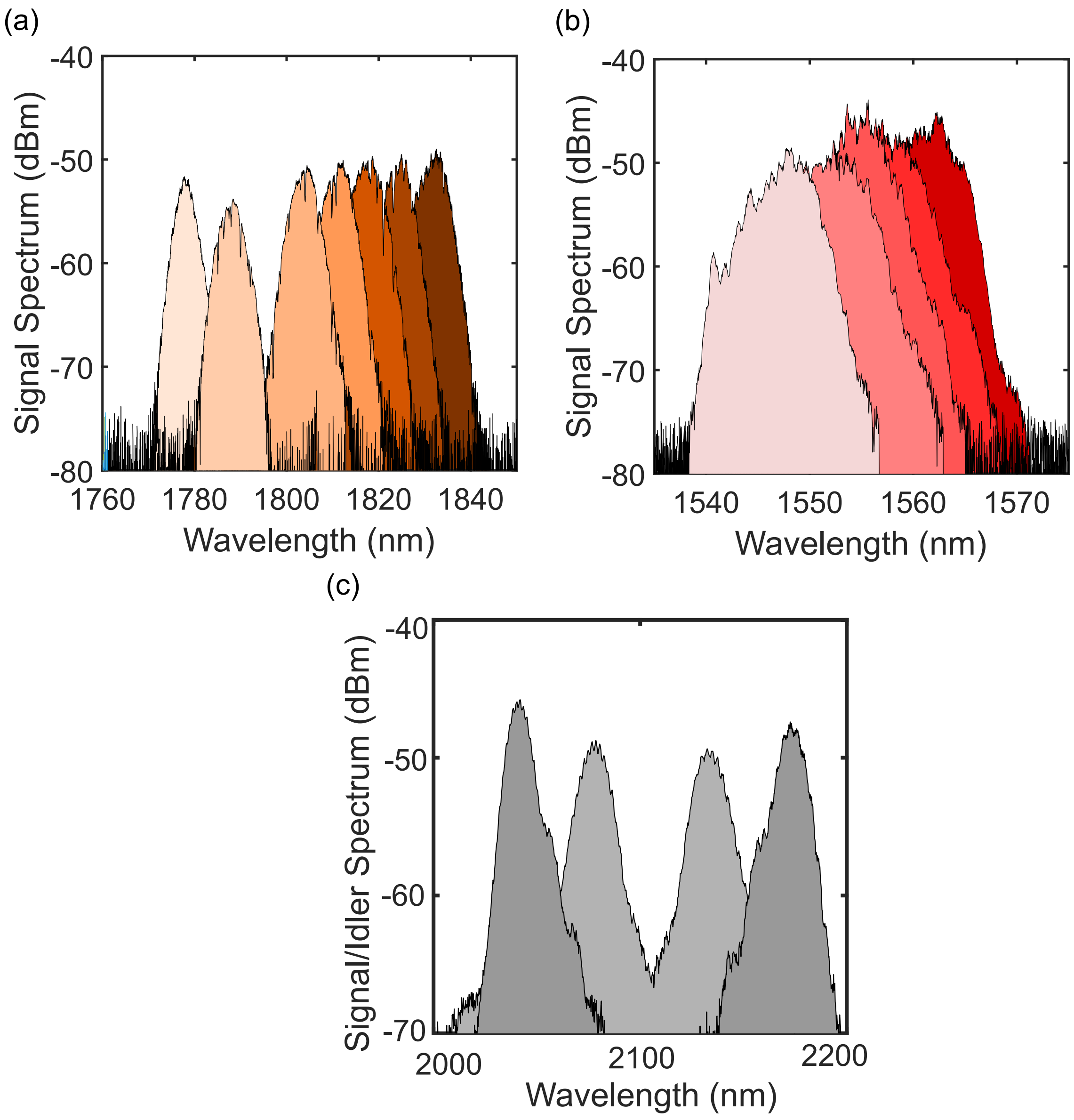

Supplementary Figure S21: Signal frequency comb tuning. a,b) Tuning of the signal frequency comb via changing the pump wavelength from two different OPOs. c) Tuning the OPO spectrum away from non-degeneracy. Two such near-degenerate spectra of the signal-idler are shown as obtained by changing the pump wavelength.

15 Future improvements and scope

The first mechanism is through tuning the phase matching. This is currently achieved experimentally by switching the poling periods. This has led to coarse tunings of the OPO outputs as shown in Fig. 2. We further establish in supplementary section 13 through numerical simulations that even with one poling period, one can achieve the whole octave tuning with temperature tuning of the poled region. However, such a temperature tuning is challenging with our current experimental setup as we explain below.

The second tuning mechanism provided by the OPO is through tuning the pump central wavelengths. For each poling period, we have shown experimentally that this mechanism can yield fine-tuning of the output spectrum. An example of such tuning is shown in Fig. 3c.

With these two tuning mechanisms, our experimental results establish the capability of the device to provide continuous tuning beyond an octave. Here, we would like to point out the experimental challenges associated with generating a single waterfall figure to cover the whole tuning spectrum.

- **Current experimental limitations in pump tuning:** This limitation pertains to our current utilization of a YDFA amplifier to boost the power levels of the pump (EO comb) and overcome the insertion loss. The YDFA has a gain window which constrained us to operate in that spectral window. Moreover, the waveshaper we used also imposed an upper cut-off wavelength of operation. Both of these combined prevented us from leveraging a wider range of pump wavelength tuning which would give us a much broader tuning range from a single OPO.
- **Current limitation on temperature tuning:** While we are confident about the temperature tuning of phase matching, systematic measurement of OPO output as a function of temperature is challenging with our current experimental setup. This is mainly because when we resort to changing the temperature of the chip, not only is the phase-matching in the periodically poled waveguide altered, but the ring cavity also experiences modification in its group index which in turn alters the FSR of the cavity. It is a cumbersome process to modify the EO comb pump for every setting of the temperature change which is required for a systematic study. Our EO comb comprises of a cascade of three phase modulators which are driven by amplified RF signal oscillating at the FSR frequency. This set of 3 amplifiers are preceded by corresponding phase delay units that are required to ensure all the phase modulators are driven in-phase. Every different setting of FSR enforces a different setting for these phase delay units and also a unique setting for the waveshaper. This is the reason we avoided the systematic study of temperature variations.
- Our lithium niobate on insulator chip uses a silica layer as an insulator. The silica is known to have an absorption band around 2.7 μm. This absorption rendered OPO threshold to be very high to see oscillation in this spectral band. Thus we won't expect to see continuous OPO output coverage in this region even though the two previous limitations are eliminated. The solution to this issue is to use a lithium niobate on a sapphire wafer. The sapphire has broadband transparency and is more suitable for mid-IR applications. Our recent devices use LNOI with sapphire as the insulating layer.

These limitations can be mitigated in our next OPO designs by using local heating components in the cavity with independent FSR tuning mechanisms. Moreover, we expect that utilization of other ps pump sources, such as our recently demonstrated mode-locked laser [26] can eliminate the limitations associated with an EO comb pump.

The pump, which is a near-IR electro-optic comb, can be incorporated into the lithium niobate chip in the future [27, 28]. With proper dispersion engineering, our OPO design can additionally achieve

large instantaneous bandwidth accompanied by significant pulse compression [14], enabling the generation of femtosecond mid-infrared frequency combs in nanophotonics. Efficient supercontinuum generation requiring only a couple of picojoules of pulse energy can then be performed using periodically-poled lithium niobate waveguides on these femtosecond pulses for subsequent f-2f self-referencing/comb stabilization [20]. Future work will involve the integration of electro-optic modulators for active locking of the OPO frequency comb. In the absence of any active feedback, the OPO output power remains stable for a long time in the laboratory environment (see Supplementary Section 7). The on-chip OPO threshold can be reduced further by improving waveguide losses and enhancing the effective nonlinear co-efficient by separately optimizing the modal overlap between the pump and the signal/idler fields for each OPO device catering to dedicated spectral bands. We estimate that an on-chip threshold for operation near degeneracy with an average power less than 500 μW (for 10 GHz repetition rate operation) is feasible. The low power requirement combined with the need for a relatively narrow pump tunability range opens the door for pumping the OPO chip with butt-coupled near-infrared diode lasers. This paves the way for a fully integrated solution for mid-IR frequency comb generation based on lithium niobate nanophotonics [23, 29, 17, 18] (see supplementary section 12).

Optimizing the coupler design can enable OPO operation with lower thresholds and higher mid-infrared comb conversion efficiency. Advanced coupler designs like the ones inspired by inverse design [30] can satisfy the simultaneous requirements of low coupling for the pump, high coupling for the signal, and optimum coupling for the idler waves, leading to conversion efficiencies even exceeding 30 %. Realizing OPO devices in lithium niobate on sapphire will give access to a wider transparency window, leading to frequency comb generation deeper into the mid-infrared [31]. Thanks to the strong parametric nonlinear interaction, it is possible to realize frequency combs with lower repetition rates ($\sim$ 1 GHz) using spiral waveguides [32] in the feedback arm of the OPO resonator which will be useful for on-chip dual-comb spectroscopy applications. The emission in the mid-infrared overlaps with important molecular rovibrational absorption lines and paves the way for novel integrated spectroscopic solutions (see Supplementary section 8).

References

[1] Ryan Hamerly, Alireza Marandi, Marc Jankowski, Martin M Fejer, Yoshihisa Yamamoto, and Hideo Mabuchi. Reduced models and design principles for half-harmonic generation in synchronously pumped optical parametric oscillators. *Physical Review A*, 94(6):063809, 2016.

[2] Matteo Conforti, Fabio Baronio, and Costantino De Angelis. Nonlinear envelope equation for broadband optical pulses in quadratic media. *Physical Review A*, 81(5):053841, 2010.

[3] JS Pelc, CR Phillips, D Chang, and C Langrock. Efficiency pedestal in quasi-phase-matching devices with random duty-cycle errors. *Optics letters*, 36(6):864–866, 2011.

[4] CR Phillips, JS Pelc, and Martin M Fejer. Parametric processes in quasi-phasematching gratings with random duty cycle errors. *JOSA B*, 30(4):982–993, 2013.

[5] MA Arbore and MM Fejer. Singly resonant optical parametric oscillation in periodically poled lithium niobate waveguides. *Optics letters*, 22(3):151–153, 1997.

[6] Andrew J Metcalf, Connor D Fredrick, Ryan C Terrien, Scott B Papp, and Scott A Diddams. 30 ghz electro-optic frequency comb spanning 300 thz in the near infrared and visible. *Optics Letters*, 44(11):2673–2676, 2019.

[7] Alexandre Parriaux, Kamal Hammani, and Guy Millot. Electro-optic frequency combs. *Advances in Optics and Photonics*, 12(1):223–287, 2020.

[8] Pan Ying, Heyun Tan, Junwei Zhang, Mingbo He, Mengyue Xu, Xiaoyue Liu, Renyou Ge, Yuntao Zhu, Chuan Liu, and Xinlun Cai. Low-loss edge-coupling thin-film lithium niobate modulator with an efficient phase shifter. *Optics letters*, 46(6):1478–1481, 2021.

[9] Ni Yao, Junxia Zhou, Renhong Gao, Jintian Lin, Min Wang, Ya Cheng, Wei Fang, and Limin Tong. Efficient light coupling between an ultra-low loss lithium niobate waveguide and an adiabatically tapered single mode optical fiber. *Optics Express*, 28(8):12416–12423, 2020.

[10] Yohei Kobayashi, Kenji Torizuka, Alireza Marandi, Robert L Byer, Richard A McCracken, Zhaowei Zhang, and Derryck T Reid. Femtosecond optical parametric oscillator frequency combs. *Journal of Optics*, 17(9):094010, 2015.

[11] Alban Douillet, J-J Zondy, Giorgio Santarelli, Ala'a Makdissi, and André Clairon. A phase-locked frequency divide-by-3 optical parametric oscillator. *IEEE Transactions on Instrumentation and Measurement*, 50(2):548–551, 2001.

[12] Kestutis Staliunas and Stefano Longhi. Cavity solitons in frequency divide-by-three optical parametric oscillator. *JOSA B*, 23(6):1124–1128, 2006.

[13] Kevin F Lee, Jie Jiang, C Mohr, J Bethge, ME Fermann, Nick Leindecker, Konstantin L Vodopyanov, Peter G Schunemann, and I Hartl. Carrier envelope offset frequency of a doubly resonant, nondegenerate, mid-infrared gaas optical parametric oscillator. *Optics letters*, 38(8):1191–1193, 2013.

[14] Arkadev Roy, Rajveer Nehra, Saman Jahani, Luis Ledezma, Carsten Langrock, Martin Fejer, and Alireza Marandi. Temporal walk-off induced dissipative quadratic solitons. *Nature Photonics*, 16(2):162–168, 2022.

[15] Jun Ye and Steven T Cundiff. *Femtosecond optical frequency comb: principle, operation and applications.* Springer Science & Business Media, 2005.

[16] Mingxiao Li, Lin Chang, Lue Wu, Jeremy Staffa, Jingwei Ling, Usman A Javid, Shixin Xue, Yang He, Raymond Lopez-Rios, Theodore J Morin, et al. Integrated pockels laser. *Nature Communications*, 13(1):1–10, 2022.

[17] Mengjie Yu, Christian Reimer, David Barton, Prashanta Kharel, Rebecca Cheng, Lingyan He, Linbo Shao, Di Zhu, Yaowen Hu, Hannah R Grant, et al. Femtosecond pulse generation via an integrated electro-optic time lens. *arXiv preprint arXiv:2112.09204*, 2021.

[18] Yaowen Hu, Mengjie Yu, Brandon Buscaino, Neil Sinclair, Di Zhu, Rebecca Cheng, Amirhassan Shams-Ansari, Linbo Shao, Mian Zhang, Joseph M Kahn, et al. High-efficiency and broadband on-chip electro-optic frequency comb generators. *Nature Photonics*, 16(10):679–685, 2022.

[19] Cheng Wang, Mian Zhang, Xi Chen, Maxime Bertrand, Amirhassan Shams-Ansari, Sethumadhavan Chandrasekhar, Peter Winzer, and Marko Lončar. Integrated lithium niobate electro-optic modulators operating at cmos-compatible voltages. *Nature*, 562(7725):101–104, 2018.

[20] Marc Jankowski, Carsten Langrock, Boris Desiatov, Alireza Marandi, Cheng Wang, Mian Zhang, Christopher R Phillips, Marko Lončar, and MM Fejer. Ultrabroadband nonlinear optics in nanophotonic periodically poled lithium niobate waveguides. *Optica*, 7(1):40–46, 2020.

[21] Yoshitomo Okawachi, Mengjie Yu, Boris Desiatov, Bok Young Kim, Tobias Hansson, Marko Lončar, and Alexander L Gaeta. Chip-based self-referencing using integrated lithium niobate waveguides. *Optica*, 7(6):702–707, 2020.

[22] O Gayer, Z Sacks, E Galun, and A Arie. Temperature and wavelength dependent refractive index equations for mgo-doped congruent and stoichiometric linbo3. *Applied Physics B*, 91(2):343–348, 2008.

[23] Luis Ledezma, Ryoto Sekine, Qiushi Guo, Rajveer Nehra, Saman Jahani, and Alireza Marandi. Intense optical parametric amplification in dispersion-engineered nanophotonic lithium niobate waveguides. *Optica*, 9(3):303–308, 2022.

[24] Arkadev Roy, Saman Jahani, Carsten Langrock, Martin Fejer, and Alireza Marandi. Spectral phase transitions in optical parametric oscillators. *Nature communications*, 12(1):1–9, 2021.

[25] Christian M Dietrich, Ihar Babushkin, José R Cardoso de Andrade, Han Rao, Ayhan Demircan, and Uwe Morgner. Higher-order dispersion and the spectral behavior in a doubly resonant optical parametric oscillator. *Optics Letters*, 45(20):5644–5647, 2020.

[26] Qiushi Guo, Ryoto Sekine, James A Williams, Benjamin K Gutierrez, Robert M Gray, Luis Ledezma, Luis Costa, Arkadev Roy, Selina Zhou, Mingchen Liu, et al. Mode-locked laser in nanophotonic lithium niobate. *arXiv preprint arXiv:2306.05314*, 2023.

[27] Mian Zhang, Brandon Buscaino, Cheng Wang, Amirhassan Shams-Ansari, Christian Reimer, Rongrong Zhu, Joseph M Kahn, and Marko Lončar. Broadband electro-optic frequency comb generation in a lithium niobate microring resonator. *Nature*, 568(7752):373–377, 2019.

[28] Camiel Op de Beeck, Felix M Mayor, Stijn Cuyvers, Stijn Poelman, Jason F Herrmann, Okan Atalar, Timothy P McKenna, Bahawal Haq, Wentao Jiang, Jeremy D Witmer, et al. Iii/v-on-lithium niobate amplifiers and lasers. *Optica*, 8(10):1288–1289, 2021.

[29] Qiushi Guo, Ryoto Sekine, Luis Ledezma, Rajveer Nehra, Devin J Dean, Arkadev Roy, Robert M Gray, Saman Jahani, and Alireza Marandi. Femtojoule femtosecond all-optical switching in lithium niobate nanophotonics. *Nature Photonics*, 16(9):625–631, 2022.

[30] Sean Molesky, Zin Lin, Alexander Y Piggott, Weiliang Jin, Jelena Vucković, and Alejandro W Rodriguez. Inverse design in nanophotonics. *Nature Photonics*, 12(11):659–670, 2018.

[31] Jatadhari Mishra, Timothy P McKenna, Edwin Ng, Hubert S Stokowski, Marc Jankowski, Carsten Langrock, David Heydari, Hideo Mabuchi, MM Fejer, and Amir H Safavi-Naeini. Mid-infrared nonlinear optics in thin-film lithium niobate on sapphire. *Optica*, 8(6):921–924, 2021.

[32] Hansuek Lee, Tong Chen, Jiang Li, Oskar Painter, and Kerry J Vahala. Ultra-low-loss optical delay line on a silicon chip. *Nature communications*, 3(1):1–7, 2012.

[33] Alireza Marandi, Zhe Wang, Kenta Takata, Robert L Byer, and Yoshihisa Yamamoto. Network of time-multiplexed optical parametric oscillators as a coherent ising machine. *Nature Photonics*, 8(12):937, 2014.
